# Supplementary material for: Class I histone deacetylases inhibition reverses memory impairment induced by acute stress in mice
Source: PLoS One. 2024 Apr 18;19(4):e0302374. doi: 10.1371/journal.pone.0302374 (PMC11025869; doi:10.1371/journal.pone.0302374)
Supplement: Attachment — Submitted filename: RESPONSE TO REVIEWERS .docx [file pone.0302374.s002.docx]

**RESPONSE TO REVIEWERS**

***Reviewer #1:*** *The paper entitled „Class I Histone Deacetylases inhibition reverses memory impairment induced by acute stress in mice” by Martínez-Pacheco et al. demonstrated the possible link between acute stress, memory disturbances and downregulation of HDAC. The topic is quite important due to the complexity and still not fully understand relationship between the stress and learning and memory processes. The manuscript is well-written but there are some deficiencies, which needs to be addressed before the acceptance. All comments are indicated below.

1. In the Introduction there should be some explanation about the rationale to use HDACs inhibitors in the study (PB and IN14), based on available literature.*

Response: Certainly, following the reviewer suggestion we have added a final paragraph in the Introduction section to address this point.

*2. In case of animals Author should write the total amount of used animals. Moreover, there should be some information about the enrichment used and handling. Authors mentioned about handling only in case of object recognition test, what about the other tests?*

Response: We have added the total amount of used animals. Information about the enrichment is now detailed in the Methods section, and indeed handling was provided to all mice before submitting them to NOR, ETM and BFLT. We have included this information as well.

*3. Why the spontaneous motor activity was measured for 5 min? Please explain.*

Response: We used spontaneous motor activity to rule out the possibility that pharmacological treatments could have any deleterious motor or motivational effect, given that adequate execution of learning tests requires the integrity of exploratory behavior. In our experience (1, 2, 3) the first 5 minutes are enough to evaluate this aspect.

*4. „Novel object recognition test” should be actually the „object recognition test”*

Response: We agree, we have corrected the test name along the text

*5. There are some shortcuts, which are not explained in the text: LTM (line 114), STM (line 126)*

Response: Certainly, we have corrected these omissions in the text

*6. Line 167-168 – please explain how this confirmation of animal’s equal preferences was performed?*

Response: In a conventional manner, it must be confirmed in each experiment that the animals do not show any preference for the objects or for their position in the box for which a pilot study is carried out with animals that will not be used again, presenting them with the objects in pairs as follows: A vs A', A vs B and A vs C.

The results are shown in Figure 1 below.

Figure 1. Investigation time of the objects used in the present study. p< 0.61; p<0.76 and p< 0.72 for panels A, B and C, respectively, T test.

We have added an explanatory paragraph of this procedure in the revised version of the manuscript. We consider it unnecessary to include this data in the manuscript since it is a standard procedure.

*7. Line 183 – Authors write „rats” whereas only mice were included in the Materials and Methods section*

Response: We have corrected this deplorable mistake

*8. „post hoc” should be written in italic*

Response: Absolutely, we have corrected this error

*9. Line 235-236 – please explain why the HDAC2 activity was evaluated only in animals from ETM test?*

Response: We chose a representative sample of 4 mice from the EPM group to perform the HDAC2 expression assay with the intention to verify its stress-induced increase as previously reported (5, 6), and to evaluate the effect of PB and IN14. We have added a paragraph alluding to it in the Methods section of the manuscript revised version.

*10. Line 269 p=0.0617 is not significant – please correct the sentence.*

Response: We have corrected this mistake

*11. Please explain why the number of animals used in case of plasma corticosterone level was only 4 per group?*

Response: To calculate the sample size, we used the power analysis formula of Charan & Kantharia, 2013 (4). Conventionally, for analyzes of blood metabolites, a sample of n=4 in triplicate is appropriate.

*12. Line 288-289 – in the Fig.4 in „vertical” – this increase doesn’t indicate to be significant.*

Response: We have modified the graph and have now presented the vertical activity using another scale so that it can be better visualized. Although the groups behave in a similar way, we have confirmed that the statistical analysis did show significant differences.

*13. Line 320 „mg/kg” with lowercase*

Response: Agreed, we have corrected this mistake

*14. Line 339 Authors write Fig.6D, which I didn’t find in the manuscript*

Response: Certainly, Fig. 6D should be 6B, we have corrected this mistake in the text

*15. In the „Discussion” section there should be more references to performed tests, like short explanation what can be measured in the specific test (please see line 139-141 – this information should be rather in the Discussion, no in the Method section)*

Response: Agreed, we have added an explanation of why we choose these three memory models in the Discussion section.

*16. Authors should include also the limitations of performed study.*

Response: We consider that the main limitation of the present study is that from our data it is difficult to infer the mechanism of action of the HDACs inhibitors we used. Our hypothesis was that pretreatment with HDACs inhibitors could mitigate the effect of the increase in corticosterone by modulating the gene expression caused by the latter; however, to our surprise, both PB and IN14 reduced the increase in corticosterone itself, which suggests its possible interaction with the hypothalamic-pituitary-adrenal axis as well, but we are not clear through what molecular mechanism. We have added a brief paragraph regarding this in the Discussion section.

*17. Figure 1 should also include the spontaneous locomotor activity test.*

Response: The spontaneous motor activity (SMA) is now included in Figure 1

*18. Figure 5C In case of PB-S after 1h – shouldn’t be there any significance vs. VEH-S?*

Response: Indeed, there is a significant difference between these two groups; we have corrected this error

*19.Figure 6B and 7A These charts are poorly legible, please try to correct them.*

Response: We have modified the figures using solid colors to make them more readable, however graphs 6B and 7A are difficult to improve because the trajectories of the groups overlap. However, we believe that what we are trying to highlight, namely, that mice behave in a very similar way (i.e., they all learn relatively well), can be easily appreciated.

*20. Figure 7B In case of PB-S– shouldn’t be there any significance vs. VEH-S?*

Response: Indeed, we have corrected this mistake

*21.Figure 8B What about the significance between VEH-S and PB-S/IN14-S?*

Response: Indeed, there is a significant difference between these two groups. We have corrected this mistake

References:

1. Castillo, L.Y. Ríos-Carrillo, J. González-Orozco, J.C. Camacho-Arroyo, I. Morin, J.-P.; Zepeda, R.C. Roldán-Roldán, G. Juvenile Exposure to BPA Alters the Estrous Cycle and Differentially Increases Anxiety-like Behavior and Brain Gene Expression in Adult Male and Female Rats. Toxics 2022, 10, 513. <https://doi.org/10.3390toxics10090513>

2. Ortiz-Pérez A, Espinosa-Raya J, Picazo O. An enriched environment and 17-beta estradiol produce similar pro-cognitive effects on ovariectomized rats. Cogn Process. 2016 Feb;17(1):15-25. doi: 10.1007/s10339-015-0746-1. Epub 2016 Feb 12. PMID: 26872959.

3. Briones-Aranda A, Castellanos-Pérez M, Villa VMV, Picazo O. Impact of Exposure to Environmental Enrichment on the Anxiety-Like Behavior of Ovariectomized Mice. Iran J Psychiatry. 2020 Jan;15(1):88-95. PMID: 32377218; PMCID: PMC7193238.

4. Charan J, Kantharia ND. How to calculate sample size in animal studies? J Pharmacol Pharmacother. 2013 Oct;4(4):303-6. doi: 10.4103/0976-500X.119726.

5. Athira KV, Madhana RM, Indu Chandran Js, Lahkar M, Sinha S, Naidu VGM. Antidepressant activity of vorinostat is associated with amelioration of oxidative stress and inflammation in a corticosterone-induced chronic stress model in mice. Behav Brain Res. 2018;344: 73-84.doi: 10.1016/j.bbr.2018.02.009. PMID: 29452193.

6. Ito K, Barnes PJ, Adcock IM. Glucocorticoid receptor recruitment of histone deacetylase 2 inhibits interleukin-1beta-induced histone H4 acetylation on lysines 8 and 12. Mol Cell Biol. 2000; 20(18): 6891-6903.doi: 10.1128/mcb.20.18.6891-6903.2000. PMID: 10958685.

*Reviewer #2: The manuscript by Martinez-Pacheco and colleagues studies the effects of acute stress on memory in mice and investigates the role of histone deacetylase 2 (HDAC2) via systemic treatment with HDAC inhibitors. Acute stress was induced by forced swimming, and the increased stress level was confirmed by plasma corticosterone level. Afterwards, three behavioral tests were conducted in independent groups and a negative effect of acute stress on short-term and long-term memory was revealed. Such memory impairment was reversed by HDAC2 inhibitors PB and IN14. In the end, the authors compared the HDAC2 levels in different treatments and found that the memory impairment is paralleled with increased HDAC2 levels. Together, this study claimed that the acute stress induces memory impairment via increased HDAC2 expression, and the memory dysfunction could be reversed by HDAC2 inhibition. The experiments are well designed and can well support their conclusions. The results are clearly represented as well. The impact of the study would be enhanced if the following comments can be addressed.

Major comments*

*1. One main conclusion of this paper is that acute stress increases HDAC2 activity, which reduces histone acetylation and memory deficits. However, the direct evidence of histone acetylation level is missing, which is essential for the conclusion. Western blots could be used to measure the acetylation levels of histone, especially H3K9 and H4K5 (Wu et al., see ref 16 in the manuscript).*

Response: We agree with the reviewer and following his suggestion we have carefully revised the manuscript and scale back our interpretations. Our intention was not to demonstrate a specific correlation between the level of histone acetylation and the impairment of learning and memory, but rather to verify in our hands that stress effectively increases the expression of HDACs (as has been previously reported) and to assess the effect of pretreatment with PB and IN14. We hope that the change made in the term activity by expression in the respective paragraphs is sufficient to avoid confusion.

*Minor comments*

*1. Line 114. The abbreviation ‘LTM tests’ should be explained by the full terminology.*

Response: Certainly, we have corrected these omissions in the text

*2. Line 458. It should be ‘antiamnestic’ instead of ‘antianmestic’.*

Response: We have corrected this mistake
